# Loneliness, Social Isolation, and Chronic Disease Outcomes in Adults: A Systematic Review

**DOI:** 10.7759/cureus.112393

**Published:** 2026-07-10

**Authors:** Afra O Ahmed, Hala B Mohamed, Amin Ahmed, Omer F Mahgoub, Mohaid Mubark Ahmed ElSarraf, Asim Ahmed, Emtenan Hamad Belowla Babeker

**Affiliations:** 1 General Medicine, Al-Yarmouk College Faculty of Medical Sciences, Khartoum, SDN; 2 General Medicine, University of Elrazi, Wad Madani, SDN; 3 General Medicine, Ministry of Public Health, Doha, QAT; 4 General Medicine, Sulaiman Al Habib Medical Group, Riyadh, SAU; 5 Family Medicine, University of Gezira, Wad Madani, SDN; 6 Medicine and Surgery, University of Gezira, Wad Madani, SDN; 7 General Medicine, University of Gezira, Wad Madani, SDN

**Keywords:** chronic disease, loneliness, social disconnection, social isolation, systematic review

## Abstract

Social disconnection, including loneliness and social isolation, is increasingly recognized as an important social determinant of adult health, but its relationship with chronic disease-related outcomes remains dispersed across disease areas and study designs. This systematic review aimed to synthesize evidence on the associations of loneliness and social isolation with chronic disease-related outcomes among adults in community, primary care, outpatient, registry-based, and population-based settings. PubMed/MEDLINE, Scopus, Web of Science, Web of Science East Mediterranean Region, Embase, Cumulative Index to Nursing and Allied Health Literature (CINAHL), PsycINFO, the Cochrane Library, Google Scholar, and reference lists were searched from database inception to May 10, 2026, for original studies reporting loneliness or social isolation in relation to cardiovascular, metabolic, respiratory, renal, frailty-related, behavioral, biological, functional, quality-of-life, morbidity, or mortality outcomes. Thirty-one studies were included and synthesized narratively because of heterogeneity in populations, exposure measures, outcomes, study designs, follow-up periods, adjustment strategies, and reported effect estimates. The included evidence was mainly observational and suggested associations between loneliness, social isolation, and a broad range of adverse chronic disease-related outcomes, with more consistent associations reported for cardiovascular disease, cardiometabolic conditions, chronic kidney disease, frailty, functional impairment, and mortality. Behavioral pathways, reduced self-management, poorer preventive behavior, lower medication adherence, and inflammatory mechanisms may partly explain these associations, although causal inference remains limited. Loneliness and social isolation appeared related but not interchangeable, as both reflect social disconnection, although loneliness is subjective, while social isolation is more objective and structural. These findings support further evaluation of social disconnection assessment in adults with or at risk of chronic disease, particularly in primary care and outpatient settings. Future research should use standardized exposure measures, repeated assessment, and stronger longitudinal, registry-linked, or intervention designs to clarify temporal directionality and determine whether improving social connection can improve chronic disease outcomes.

## Introduction and background

Loneliness and social isolation are increasingly recognized as important public health concerns with relevance to adult health and chronic disease prevention. The World Health Organization has emphasized social connection as a major determinant of health and has highlighted the health, social, and economic consequences of loneliness and social isolation [1]. These forms of social disconnection may influence chronic disease prevention and long-term disease risk through health behaviors, self-management, healthcare engagement, and stress-related responses. This concern is especially relevant in the context of noncommunicable diseases because long-term illness is shaped not only by biological risk but also by social conditions that affect prevention, treatment, and daily self-management. Chronic diseases, including cardiovascular disease, diabetes, chronic respiratory disease, chronic kidney disease, and other long-term conditions, remain major contributors to morbidity and mortality worldwide [2]. These conditions are influenced by genetic, physiological, behavioral, environmental, and social determinants, making social disconnection a plausible social factor associated with chronic disease risk, progression, and prognosis [2].

In this review, social disconnection is used as an umbrella term that includes both loneliness and social isolation. Loneliness refers to the subjective feeling that social or emotional connection is inadequate or unsatisfying, whereas social isolation refers to a more objective lack of social contact, participation, or network engagement [3]. This distinction is important because individuals may be socially isolated without feeling lonely or may feel lonely despite regular social contact [3]. Previous evidence has shown that stronger social relationships are associated with lower mortality risk, while loneliness, social isolation, and living alone are associated with higher premature mortality [4,5]. Cardiovascular disease is one of the most studied outcomes in this field, with longitudinal evidence showing that loneliness and social isolation are associated with increased risk of coronary heart disease and stroke [6].

Several mechanisms may help explain these associations. Behavioral pathways may include reduced physical activity, unhealthy lifestyle patterns, and weaker healthcare engagement. Psychological pathways may include stress, depressive symptoms, and reduced motivation for self-care. Biological pathways may include neuroendocrine dysregulation, inflammation, impaired immune regulation, vascular dysfunction, and metabolic dysregulation [7-9]. These pathways suggest that loneliness and social isolation may be associated with chronic disease-related outcomes through overlapping behavioral, psychological, and biological mechanisms.

Evidence beyond cardiovascular disease remains broad but fragmented. Prior reviews have linked loneliness and social isolation with several adult health outcomes, including mortality, mental health, cognitive decline, physical function, quality of life, and health service use. This evidence is especially relevant to older and community-dwelling adults because chronic disease, functional limitation, and reduced social participation often overlap in this population [10]. Chronic disease may also increase loneliness and social isolation by limiting mobility, independence, and social participation, creating a possible bidirectional relationship between social disconnection and long-term illness [11]. This bidirectional concern is further supported by evidence from older community populations showing that both social isolation and loneliness are associated with all-cause mortality, although social isolation may retain a stronger independent association after adjustment for demographic, socioeconomic, and health factors [12].

Despite the growing literature, evidence on social disconnection and chronic disease-related outcomes remains scattered across disease areas, study designs, and outcome measures. Existing evidence includes cardiovascular, metabolic, respiratory, renal, frailty-related, behavioral, biological, quality-of-life, morbidity, and mortality outcomes, but the consistency and scope of these associations have not been clearly synthesized in a focused review of community-dwelling adult populations. This systematic review addresses this gap by summarizing evidence on loneliness and social isolation in adults from community, primary care, outpatient, registry-based, and population-based settings. This focus was chosen to capture social disconnection in everyday adult chronic disease care and population-based contexts, rather than in inpatient, institutionalized, nursing-home-only, or psychiatric-only settings. The aim of this systematic review is to synthesize evidence on the association of loneliness and social isolation with chronic disease-related outcomes among community-dwelling adults.

## Review

This systematic review synthesized evidence on the association of social disconnection with chronic disease-related outcomes among adults in community, primary care, outpatient, registry-based, and population-based settings. Social disconnection was used as an umbrella term that included loneliness and social isolation. The review was conducted according to the Preferred Reporting Items for Systematic Reviews and Meta-Analyses 2020 statement where applicable [13]. This review was not prospectively registered, and no review protocol was published before the review was conducted.

The review question was: among adults in community, primary care, outpatient, registry-based, or population-based settings, is social disconnection associated with chronic disease-related outcomes, including cardiovascular, metabolic, respiratory, renal, frailty-related, behavioral, biological, functional, quality-of-life, morbidity, and mortality outcomes? The population, intervention/exposure, comparator, outcomes (PICO)/population, exposure, comparator, outcomes (PECO) framework used to structure the review question and eligibility criteria is shown in Table 1.

**Table 1:** PICO/PECO framework used in this systematic review. CKD: chronic kidney disease; COPD: chronic obstructive pulmonary disease; NAFLD: non-alcoholic fatty liver disease; PECO: population, exposure, comparator, outcomes; PICO: population, intervention/exposure, comparator, outcomes.

Element	Definition used in this review
Population	Adults aged 18 years or older. Eligible settings included community, primary care, outpatient, registry-based, and population-based settings.
Intervention/exposure	Loneliness, social isolation, or directly measured social disconnection. Social support, social participation, social relationships, and social connectedness were considered only when analyzed alongside loneliness or social isolation.
Comparator	Adults without loneliness or social isolation, adults with lower loneliness or isolation scores, or adults with stronger social participation, social relationships, or social connection, depending on study design.
Outcomes	Chronic disease-related outcomes, including cardiovascular disease, diabetes, metabolic syndrome, cardiometabolic multimorbidity, non-alcoholic fatty liver disease, chronic respiratory disease, chronic obstructive pulmonary disease, asthma, chronic kidney disease, frailty, malnutrition risk, medication adherence, preventive behavior, quality of life, inflammatory markers, functional impairment, morbidity, and mortality.

Studies were eligible if they included adults aged 18 years or older. Eligible settings included community, primary care, outpatient, registry-based, and population-based settings. Studies also had to measure loneliness or social isolation as a main exposure and report at least one chronic disease-related outcome. Eligible outcomes included disease incidence, disease progression, disease-related mortality, cardiovascular outcomes, metabolic outcomes, respiratory outcomes, renal outcomes, frailty-related outcomes, functional outcomes, behavioral outcomes, quality-of-life outcomes, biological or inflammatory markers, morbidity, and mortality. Eligible study designs included prospective and retrospective cohort studies, longitudinal panel studies, cross-sectional studies, qualitative studies, mixed-methods studies, registry-linked observational studies, and Mendelian randomization studies, when relevant observational evidence was also provided. Only English-language, peer-reviewed original research articles with extractable data were included. Grey literature and preprints were not included.

Studies were excluded if they were pediatric-only studies; inpatient-only, nursing-home-only, or institutionalized-only studies without relevant community or outpatient data; psychiatric-only studies without chronic disease-related outcomes; studies measuring only social support without loneliness or social isolation; or prevalence-only studies without health outcomes. Reviews, editorials, commentaries, protocols, conference abstracts without sufficient data, and articles without original analyzable data were also excluded.

The search strategy was designed for PubMed/MEDLINE, Scopus, Web of Science, Web of Science East Mediterranean Region (WOS-EMR), Embase, Cumulative Index to Nursing and Allied Health Literature (CINAHL), PsycINFO, and the Cochrane Library. Searches were conducted from database inception to May 10, 2026. Google Scholar and reference-list screening were used as supplementary sources to identify additional relevant published studies. Google Scholar was used for targeted supplementary searching rather than as a fully systematic database search. Additional targeted searching was performed through DOI links, publisher pages, PubMed records, and direct article pages when needed to verify study details, retrieve bibliographic information, or clarify missing information. The search combined terms for loneliness and social isolation with terms for chronic disease-related outcomes and adult community-based settings. The search strategy was shaped to keep the review close to chronic disease-related outcomes and to avoid unrelated patient-experience, psychiatric-only, social support-only, and inpatient-only literature. The search strings used across the main databases and supplementary sources are summarized in Table 2. 

**Table 2:** Search strings used in the systematic review. CINAHL: Cumulative Index to Nursing and Allied Health Literature; COPD: chronic obstructive pulmonary disease; NAFLD: non-alcoholic fatty liver disease; WOS-EMR: Web of Science East Mediterranean Region.

Source	Search string
PubMed/MEDLINE	("loneliness" OR "social isolation" OR "social disconnection" OR "social connectedness" OR "social participation" OR "social relationships") AND ("chronic disease" OR "cardiovascular disease" OR "heart disease" OR stroke OR diabetes OR "type 2 diabetes" OR "metabolic syndrome" OR "cardiometabolic multimorbidity" OR multimorbidity OR COPD OR asthma OR "chronic kidney disease" OR frailty OR "non-alcoholic fatty liver disease" OR NAFLD OR mortality OR "medication adherence" OR "preventive behavior" OR inflammation OR "quality of life") AND (adult OR adults OR "older adults" OR "middle-aged" OR "community-dwelling" OR "primary care" OR outpatient OR "population-based")
Scopus	TITLE-ABS-KEY (loneliness OR "social isolation" OR "social disconnection" OR "social connectedness" OR "social participation" OR "social relationships") AND TITLE-ABS-KEY ("chronic disease" OR "cardiovascular disease" OR "heart disease" OR stroke OR diabetes OR "type 2 diabetes" OR "metabolic syndrome" OR "cardiometabolic multimorbidity" OR multimorbidity OR COPD OR asthma OR "chronic kidney disease" OR frailty OR "non-alcoholic fatty liver disease" OR NAFLD OR mortality OR "medication adherence" OR "preventive behavior" OR inflammation OR "quality of life") AND TITLE-ABS-KEY (adult OR adults OR "older adults" OR "middle-aged" OR "community-dwelling" OR "primary care" OR outpatient OR "population-based")
Web of Science	TS=(loneliness OR "social isolation" OR "social disconnection" OR "social connectedness" OR "social participation" OR "social relationships") AND TS=("chronic disease" OR "cardiovascular disease" OR "heart disease" OR stroke OR diabetes OR "type 2 diabetes" OR "metabolic syndrome" OR "cardiometabolic multimorbidity" OR multimorbidity OR COPD OR asthma OR "chronic kidney disease" OR frailty OR "non-alcoholic fatty liver disease" OR NAFLD OR mortality OR "medication adherence" OR "preventive behavior" OR inflammation OR "quality of life") AND TS=(adult OR adults OR "older adults" OR "middle-aged" OR "community-dwelling" OR "primary care" OR outpatient OR "population-based")
WOS-EMR	("loneliness" OR "social isolation" OR "social disconnection" OR "social connectedness" OR "social participation" OR "social relationships") AND ("chronic disease" OR "cardiovascular disease" OR "heart disease" OR stroke OR diabetes OR "type 2 diabetes" OR "metabolic syndrome" OR "cardiometabolic multimorbidity" OR multimorbidity OR COPD OR asthma OR "chronic kidney disease" OR frailty OR "non-alcoholic fatty liver disease" OR NAFLD OR mortality OR "medication adherence" OR "preventive behavior" OR inflammation OR "quality of life") AND (adult OR adults OR "older adults" OR "middle-aged" OR "community-dwelling" OR "primary care" OR outpatient OR "population-based")
Embase	('loneliness' OR 'social isolation' OR 'social disconnection' OR 'social connectedness' OR 'social participation' OR 'social relationships') AND ('chronic disease' OR 'cardiovascular disease' OR 'heart disease' OR stroke OR diabetes OR 'type 2 diabetes' OR 'metabolic syndrome' OR 'cardiometabolic multimorbidity' OR multimorbidity OR COPD OR asthma OR 'chronic kidney disease' OR frailty OR 'non-alcoholic fatty liver disease' OR NAFLD OR mortality OR 'medication adherence' OR 'preventive behavior' OR inflammation OR 'quality of life') AND (adult OR adults OR 'older adults' OR 'middle-aged' OR 'community-dwelling' OR 'primary care' OR outpatient OR 'population-based')
CINAHL	("loneliness" OR "social isolation" OR "social disconnection" OR "social connectedness" OR "social participation" OR "social relationships") AND ("chronic disease" OR "cardiovascular disease" OR "heart disease" OR stroke OR diabetes OR "type 2 diabetes" OR "metabolic syndrome" OR "cardiometabolic multimorbidity" OR multimorbidity OR COPD OR asthma OR "chronic kidney disease" OR frailty OR "non-alcoholic fatty liver disease" OR NAFLD OR mortality OR "medication adherence" OR "preventive behavior" OR inflammation OR "quality of life") AND (adult OR adults OR "older adults" OR "middle-aged" OR "community-dwelling" OR "primary care" OR outpatient OR "population-based")
PsycINFO	("loneliness" OR "social isolation" OR "social disconnection" OR "social connectedness" OR "social participation" OR "social relationships") AND ("chronic disease" OR "cardiovascular disease" OR "heart disease" OR stroke OR diabetes OR "type 2 diabetes" OR "metabolic syndrome" OR "cardiometabolic multimorbidity" OR multimorbidity OR COPD OR asthma OR "chronic kidney disease" OR frailty OR "non-alcoholic fatty liver disease" OR NAFLD OR mortality OR "medication adherence" OR "preventive behavior" OR inflammation OR "quality of life") AND (adult OR adults OR "older adults" OR "middle-aged" OR "community-dwelling" OR "primary care" OR outpatient OR "population-based")
Cochrane Library	("loneliness" OR "social isolation" OR "social disconnection" OR "social connectedness" OR "social participation" OR "social relationships") AND ("chronic disease" OR "cardiovascular disease" OR "heart disease" OR stroke OR diabetes OR "type 2 diabetes" OR "metabolic syndrome" OR "cardiometabolic multimorbidity" OR multimorbidity OR COPD OR asthma OR "chronic kidney disease" OR frailty OR "non-alcoholic fatty liver disease" OR NAFLD OR mortality OR "medication adherence" OR "preventive behavior" OR inflammation OR "quality of life")
Supplementary targeted search	("loneliness" OR "social isolation") AND ("cardiovascular disease" OR diabetes OR "chronic kidney disease" OR COPD OR asthma OR frailty OR multimorbidity OR mortality) AND ("community-dwelling adults" OR "older adults" OR "population-based cohort")

Study selection process

Retrieved records were imported into a reference-management workflow, and duplicates were removed before screening. Two reviewers independently screened titles and abstracts against the eligibility criteria, and potentially eligible studies were then assessed in full text by the same reviewers. Studies were retained only when they measured loneliness or social isolation and reported an eligible chronic disease-related outcome. When the same cohort appeared in more than one article, each article was included only if it addressed a distinct outcome, exposure pattern, subgroup, or analytic question. Disagreements or uncertainties were resolved through discussion and rechecking against the eligibility criteria; unresolved disagreements were referred to a third reviewer.

Two reviewers used a structured extraction form, and the collected information was checked for consistency before synthesis. Extracted items included study ID, author and year, country or cohort, study design, setting, population, sample size, age group, exposure type, exposure measurement tool, comparator or reference group, outcome domain, outcome definition, follow-up duration, effect estimate, confidence interval where available, P value where available, key adjusted covariates, main finding, and synthesis domain. For qualitative studies, the extraction focused on the setting, population, data source, qualitative approach, key themes, relevance to loneliness or social isolation, and contribution to the interpretation of chronic disease. When key information was unclear or missing, the article was rechecked through the full text, publisher page, PubMed record, or DOI-linked record. When missing information could not be confirmed, it was recorded as not reported.

A narrative synthesis was performed because the included studies were clinically and methodologically heterogeneous, particularly in relation to populations, exposure measures, and outcome definitions. Studies were grouped by outcome domain, including cardiovascular disease, metabolic and cardiometabolic outcomes, respiratory disease, chronic kidney disease, and other domains such as frailty and functional impairment, preventive behavior and medication adherence, quality of life, biological or inflammatory pathways, morbidity, and mortality. Within each domain, findings were compared according to exposure type, study design, direction of association, adjustment strength, temporality, outcome type, and risk of bias. Loneliness and social isolation were interpreted separately whenever possible because loneliness represents subjective social disconnection, whereas social isolation represents objective or structural lack of social contact, participation, or network connection. A formal Grading of Recommendations Assessment, Development and Evaluation (GRADE) certainty assessment was not performed.

Meta-analysis was not performed because the included studies differed substantially in population source, exposure measurement, outcome definition, study design, follow-up duration, adjustment variables, and reported effect measures. Adjusted effect estimates, confidence intervals, P values, and key covariates were considered where reported, but these data were not consistently available or directly comparable across all included studies. Loneliness was measured using different tools, including multi-item University of California, Los Angeles (UCLA)-based scales and single-item measures. Social isolation was measured using varying composite indicators, including living arrangement, social contact, social participation, marital status, and network characteristics. The outcomes were also too diverse for valid pooling. They included cardiovascular disease, stroke, asthma, chronic obstructive pulmonary disease mortality, chronic kidney disease, diabetes, metabolic syndrome, non-alcoholic fatty liver disease, cardiometabolic multimorbidity, frailty, malnutrition risk, medication adherence, preventive behavior, quality of life, inflammation, functional impairment, morbidity, and mortality. Because these outcomes did not represent a single comparable endpoint, a pooled effect estimate would have been clinically difficult to interpret and statistically inappropriate. Therefore, structured narrative synthesis was considered more suitable.

Risk of bias was assessed by two reviewers using design-appropriate tools, with disagreements resolved by discussion or referral to a third reviewer. Cohort and registry-linked observational studies were assessed using the Newcastle-Ottawa Scale, with attention to selection, comparability, and outcome assessment. The Newcastle-Ottawa Scale assesses the quality of non-randomized studies using a star-based approach across selection, comparability, and exposure or outcome domains [14]. Cross-sectional studies were assessed using the Joanna Briggs Institute critical appraisal checklist for analytical cross-sectional studies. The Joanna Briggs Institute tools assess the trustworthiness, relevance, and results of published studies, and the analytical cross-sectional checklist considers key domains such as eligibility criteria, measurement quality, confounding, and statistical analysis [15]. Qualitative evidence was assessed using the Critical Appraisal Skills Program qualitative checklist. This checklist appraises qualitative studies across domains including clarity of aims, appropriateness of methodology, research design, recruitment, data collection, reflexivity, ethical issues, analysis, findings, and value of the research [16]. Mendelian randomization evidence, when present, was interpreted cautiously with attention to the assumptions of instrument relevance, independence, and exclusion restriction. Risk-of-bias judgments were categorized as low, low-to-moderate, moderate, moderate-to-high, or high according to study design, exposure measurement, outcome ascertainment, confounding control, follow-up, and suitability of analysis. Studies were generally judged to have low risk of bias when they used longitudinal or registry-linked designs, clear exposure measurement, valid outcome ascertainment, adequate adjustment for confounding, and appropriate follow-up. Studies were generally judged to have a moderate risk of bias when they had some concerns related to self-reported exposure, limited adjustment, attrition, or indirect exposure measurement. Studies were judged to have a higher risk of bias when major concerns were present regarding temporality, confounding, exposure or outcome measurement, sample size, or study design.

Results 

Study Selection

**Figure 1:**
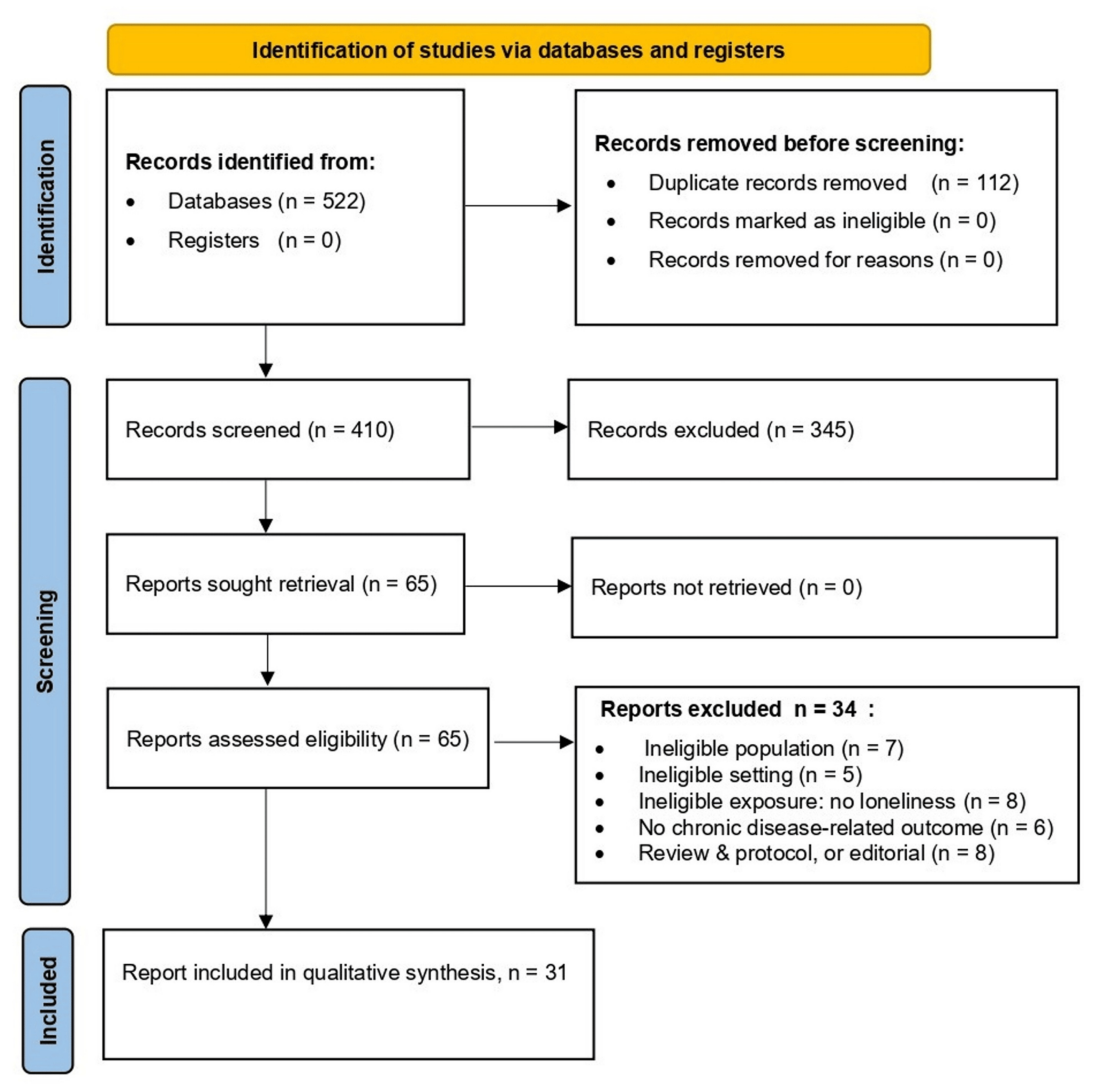
PRISMA flow diagram of study selection. The diagram shows the study identification and selection process. A total of 522 records were identified from databases, and 112 duplicate records were removed before screening. After screening 410 records, 345 were excluded. Sixty-five reports were sought and assessed for eligibility, and 34 reports were excluded for predefined reasons, including ineligible population, ineligible setting, ineligible exposure, absence of chronic disease-related outcomes, or non-original article type. Thirty-one studies were included in the final qualitative synthesis.

The database and supplementary searches identified 522 records. After removal of 112 duplicate records, 410 records were screened by title and abstract. Of these, 345 records were excluded because they were not relevant to the review question, did not include loneliness or social isolation as eligible exposures, did not report chronic disease-related outcomes, or were not original research articles. The full texts of 65 articles were assessed for eligibility. After full-text assessment, 34 articles were excluded for reasons including ineligible population, ineligible setting, ineligible exposure, absence of chronic disease-related outcomes, review or commentary design, insufficient data, or duplicate cohort information without a distinct outcome or analysis. Thirty-one studies were included in the final qualitative synthesis [17-47]. The study selection process is summarized in Figure 1.

*Study Characteristics* 

Thirty-one studies were included in the qualitative synthesis [17-47]. The included evidence covered adults in community, primary care, outpatient, registry-based, and population-based settings. Study designs included prospective cohort studies, retrospective cohort analyses, longitudinal panel studies, cross-sectional studies, qualitative secondary analysis, mechanistic observational studies, and one study combining prospective cohort analysis with Mendelian randomization. The included studies mainly examined loneliness and social isolation, with related social connection constructs considered when they helped interpret wider social disconnection in relation to chronic disease-related outcomes. The main outcome domains were cardiovascular disease, metabolic and cardiometabolic outcomes, respiratory disease, chronic kidney disease, frailty, malnutrition risk, preventive behavior, medication adherence, quality of life, inflammation, functional impairment, morbidity, and mortality. Because the included studies differed in exposure definitions, outcome domains, follow-up periods, adjustment strategies, and effect measures, findings were synthesized narratively. The main characteristics of the included studies are summarized in Table 3. 

**Table 3:** Included studies and main extracted findings. CHARLS: China Health and Retirement Longitudinal Study; CI: confidence interval; COPD: chronic obstructive pulmonary disease; CMM: cardiometabolic multimorbidity; CMD: cardiometabolic disease; CRP: C-reactive protein; CVD: cardiovascular disease; ELSA: English Longitudinal Study of Ageing; FRS: Framingham Risk Score; HR: hazard ratio; NAFLD: non-alcoholic fatty liver disease; NHANES: National Health and Nutrition Examination Survey; OR: odds ratio; suPAR: soluble urokinase plasminogen activator receptor; VHD: valvular heart disease.

Study	Design and setting	Population	Exposure	Main outcome	Key finding	Main statistical estimate	Adjustment or reporting note
Ida et al. 2023 [17]	Cross-sectional outpatient study, Japan	163 diabetic outpatients aged 65 years or older	Loneliness	Malnutrition risk	Loneliness was associated with higher malnutrition risk, adjusted OR 3.81, 95% CI 1.27-11.39.	Adjusted OR 3.81, 95% CI 1.27-11.39, P=0.017.	Adjusted estimate for loneliness and malnutrition risk.
Kurpas et al. 2016 [18]	Primary care observational study, Poland	582 patients with chronic respiratory and non-respiratory disease	Social relationships	Respiratory disease care and quality of life	Better social relationships were linked with fewer nurse interventions, fewer hospitalizations, and fewer chronic diseases.	No comparable OR/HR was reported for the extracted outcome.	Qualitative summary retained because the exposure was social relationships rather than direct loneliness or social isolation.
Hu et al. 2021 [19]	Cross-sectional older-adult study	Older adults	Social isolation, loneliness, and social support	Cardiovascular disease risk factors	Social constructs showed different associations with cardiovascular risk factors, supporting separate assessment of loneliness and isolation.	Among men, loneliness was associated with greater Framingham Risk Score (beta=0.03, P<0.001).	Cross-sectional cardiovascular risk-factor analysis.
Shankar et al. 2011 [20]	Observational English Longitudinal Study of Ageing analysis, England	Older adults	Loneliness and social isolation	Health behaviors and biomarkers	Loneliness and isolation were associated with unhealthy behaviors; isolation was also linked with blood pressure, C-reactive protein, and fibrinogen.	No single comparable OR/HR was reported for behavioral and biomarker outcomes.	Behavioral and biomarker pathway evidence.
Valtorta et al. 2018 [21]	Longitudinal English Longitudinal Study of Ageing cohort, England	Older adults	Loneliness and social isolation	Cardiovascular disease risk	Loneliness was associated with increased cardiovascular disease risk.	Loneliness and CVD: OR 1.27, 95% CI 1.01-1.57; social isolation was not associated with CVD.	Longitudinal ELSA analysis.
Chen et al. 2024 [22]	Prospective UK Biobank cohort	18,258 adults aged 38-73 years	Persistent or changing loneliness and isolation	Cardiovascular disease, mortality, and cardiac function	Persistent loneliness and isolation were associated with incident cardiovascular disease, mortality, and poorer cardiac function.	Persistent social isolation: CVD HR 1.17, 95% CI 1.03-1.33; all-cause mortality HR 1.42, 95% CI 1.12-1.81; CVD mortality HR 1.53, 95% CI 1.05-2.23. Persistent loneliness: CVD HR 1.13, 95% CI 1.00-1.27; all-cause mortality HR 1.28, 95% CI 1.02-1.61; CVD mortality HR 1.52, 95% CI 1.06-2.18.	Multivariable Cox regression adjusted for sociodemographic, lifestyle, and health factors.
Yue et al. 2026 [23]	Retrospective NHANES cohort, United States	1,726 adults with chronic obstructive pulmonary disease	Social isolation	All-cause, cardiovascular, and chronic lower respiratory disease mortality	Social isolation was associated with higher mortality, partly mediated by inflammatory ratios.	Social isolation was associated with higher all-cause, cardiovascular, and chronic lower respiratory disease mortality.	Retrospective NHANES cohort with mediation by systemic inflammatory markers.
Cao et al. 2026 [24]	Population-based cohort	Adults without baseline valvular disease	Social disconnection	Degenerative valvular heart disease	Social disconnection was associated with incident degenerative valvular heart disease.	Highest loneliness level: HR 1.19, 95% CI 1.09-1.28 for degenerative VHD; social isolation was not significant.	Population-based cohort analysis.
Zhang et al. 2026 [25]	Prospective UK Biobank cohort	Individuals with metabolic syndrome	Social isolation and loneliness	All-cause and cause-specific mortality	Social isolation and loneliness were associated with higher mortality, especially when both were present.	Social isolation and loneliness were associated with increased all-cause and cardiovascular mortality among individuals with metabolic syndrome.	Prospective UK Biobank cohort; effect direction was consistent across mortality endpoints.
Zhang et al. 2026 [26]	UK Biobank cohort and Mendelian randomization study	400,184 asthma-free adults	Loneliness and social isolation	Adult-onset asthma	Loneliness was associated with adult-onset asthma; social isolation was not significant after full adjustment.	Loneliness and adult-onset asthma: HR 1.27, 95% CI 1.15-1.39; social isolation was not significant after full adjustment.	Fully adjusted cohort model with supporting Mendelian randomization analysis.
Xie et al. 2026 [27]	Longitudinal CHARLS cohort, China	8,046 adults aged 45 years or older free of cardiovascular disease	Loneliness	Incident cardiovascular disease, heart disease, and stroke	Loneliness was associated with increased incident cardiovascular disease, heart disease, and stroke.	Loneliness was associated with increased incident cardiovascular disease, heart disease, and stroke.	Longitudinal CHARLS cohort analysis.
Qin et al. 2026 [28]	Prospective English Longitudinal Study of Ageing cohort, England	7,845 adults aged 50 years or older	Social asymmetry	Cardiovascular disease, chronic obstructive pulmonary disease, dementia, and mortality	Social vulnerability was associated with higher mortality, cardiovascular disease, and chronic obstructive pulmonary disease risk.	Higher social asymmetry: CVD HR 1.06, 95% CI 1.02-1.10; all-cause mortality HR 1.04, 95% CI 1.01-1.06. Social vulnerability: all-cause mortality HR 1.13, 95% CI 1.04-1.22; CVD HR 1.16, 95% CI 1.04-1.30; COPD HR 1.21, 95% CI 1.04-1.42.	Longitudinal ELSA analysis.
Kelechi et al. 2026 [29]	Social genomics observational study	Adults with chronic lower extremity ulcers	Loneliness	Systemic inflammation	Loneliness was linked with inflammatory biology in adults with chronic wounds.	Lonely group showed elevated proinflammatory gene expression over two visits (P=0.010).	Mechanistic biomarker outcome; no comparable clinical OR/HR.
Wang and Wang, 2026 [30]	Two prospective East Asian cohorts	9,381 Chinese and 5,052 Korean adults	Change in loneliness	Cardiometabolic multimorbidity	Persistent loneliness showed the strongest association with cardiometabolic multimorbidity.	CHARLS: initiated loneliness aHR 1.42, 95% CI 1.14-1.78; relieved loneliness aHR 1.40, 95% CI 1.16-1.70; persistent loneliness aHR 2.03, 95% CI 1.64-2.51. KLoSA: relieved loneliness aHR 1.72, 95% CI 1.07-2.76; persistent loneliness aHR 1.86, 95% CI 1.21-2.88.	Multivariable Cox regression in two East Asian cohorts.
Hackett et al. 2020 [31]	Longitudinal English Longitudinal Study of Ageing cohort, England	4,112 diabetes-free adults	Loneliness	Incident type 2 diabetes	Loneliness predicted type 2 diabetes independent of demographic, behavioral, metabolic, depressive, and social factors.	Final model: HR 1.41, 95% CI 1.04-1.90, P=0.027.	Adjusted for demographic, socioeconomic, behavioral, cardiometabolic, depressive, living-alone, and social-isolation variables.
Sluiter et al. 2024 [32]	Secondary qualitative analysis	Patients with chronic kidney disease and caregivers	Loneliness and social isolation	Lived experience in chronic kidney disease	Chronic kidney disease restricted social participation and deepened loneliness and isolation.	Not applicable.	Qualitative secondary analysis; no effect estimate expected.
Yamaguchi et al. 2020 [33]	Cross-sectional community study, Japan	57 community-dwelling older adults	Social isolation and loneliness	Noncommunicable disease preventive behaviors	Social disconnection was associated with poorer diet, smoking-related behaviors, and lower physical activity.	No single comparable OR/HR was reported; associations were described between social isolation, loneliness, and noncommunicable disease preventive behaviors.	Small cross-sectional behavioral pathway study.
Lu et al. 2020 [34]	Cross-sectional chronic disease study	Older adults with chronic diseases	Social isolation and loneliness	Medication adherence	Social isolation was linked with poorer adherence through reduced support and increased loneliness.	No comparable OR/HR was reported for the medication-adherence mediation outcome.	Cross-sectional serial mediation analysis.
Naito et al. 2021 [35]	Multinational prospective cohort	Adults from 20 countries	Social isolation	Mortality and morbidity	Social isolation was associated with increased mortality and morbidity across global settings.	Mortality HR 1.26, 95% CI 1.17-1.36; stroke HR 1.23, 95% CI 1.07-1.40; CVD HR 1.15, 95% CI 1.05-1.25; pneumonia HR 1.22, 95% CI 1.09-1.37.	Large multinational prospective cohort; no association with cancer.
Ge et al. 2022 [36]	Panel data analysis, Singapore	606 adults aged 60 years or older	Social isolation, social participation, and loneliness	Frailty	Loneliness was associated with higher frailty, while social participation was protective.	Feeling lonely: OR 2.90, 95% CI 1.44-5.84; social participation: OR 0.96, 95% CI 0.93-0.99; social isolation was not associated with frailty.	Fixed-effects ordinal logistic regression adjusted for time-varying sociodemographic, lifestyle, and health-related factors.
Freak-Poli et al. 2021 [37]	Prospective older-adult cohort	Healthy community-dwelling older adults	Social isolation, loneliness, and social support	Cardiovascular disease incidence and mortality	Social isolation and low support appeared more strongly associated with cardiovascular outcomes than loneliness.	Incident CVD: social isolation HR 1.66, P=0.04; low social support HR 2.05, P=0.002; loneliness HR 1.40, P=0.10. Ischemic stroke HR range 1.73-3.16 for poor social health measures.	Cardiovascular outcomes reported for multiple social health measures.
Christiansen et al. 2021 [38]	Prospective registry-linked cohort, Denmark	Adult population survey participants	Loneliness and social isolation	Cardiovascular disease, chronic obstructive pulmonary disease, type 2 diabetes, and cancer	Loneliness and isolation were associated with later cardiovascular disease and type 2 diabetes.	Loneliness and social isolation were independently associated with cardiovascular disease and type 2 diabetes within five years; no significant association was reported for COPD or cancer.	Registry-linked cohort; associations were partly explained by baseline psychological and behavioral factors.
Ye et al. 2024 [39]	Longitudinal European cohort	1,735 community-dwelling older adults	Loneliness	Frailty	Social loneliness was associated with higher overall, physical, and social frailty.	No comparable OR/HR was reported for frailty-domain associations.	Longitudinal analysis of bidirectional associations across frailty domains.
Whisman, 2010 [40]	Population-based study	Middle-aged and older adults	Loneliness	Metabolic syndrome	Loneliness was associated with metabolic syndrome and central obesity.	Each 1-unit increase in loneliness was associated with approximately 10% higher odds of metabolic syndrome.	Population-based metabolic syndrome study.
Xiao et al. 2024 [41]	Prospective UK Biobank cohort	Adults with or without baseline cardiometabolic disease	Social isolation and loneliness	Cardiometabolic disease and multimorbidity	Social isolation and loneliness were associated with disease onset and progression to multimorbidity.	Most isolated vs least isolated: CMD HR 1.07, 95% CI 1.03-1.11; CMM HR 1.24, 95% CI 1.12-1.36 in stage I; CMM HR 1.14, 95% CI 1.05-1.23 in stage II. Highest loneliness was associated with about 20% higher CMD risk and 29% higher CMM risk in stage I.	Multivariable UK Biobank analysis.
Miao et al. 2025 [42]	Prospective UK Biobank cohort	Adults free of non-alcoholic fatty liver disease at baseline	Loneliness and social isolation	Incident non-alcoholic fatty liver disease	Loneliness and isolation were associated with incident non-alcoholic fatty liver disease, partly explained by lifestyle, depression, and inflammation.	Loneliness HR 1.22 and social isolation HR 1.13 for incident NAFLD.	Prospective UK Biobank cohort; mediation by lifestyle, depression, and inflammation was reported.
Tang et al. 2024 [43]	Cohort study in diabetes	Adults with diabetes	Loneliness and social isolation	Incident chronic kidney disease	Loneliness was associated with increased chronic kidney disease risk among patients with diabetes.	Higher loneliness scale was associated with 25% higher CKD risk; feeling lonely versus not lonely was associated with 22% higher CKD risk.	Fully adjusted model; social isolation was not significant.
Guo et al. 2026 [44]	Population-based cohort	Adult population	Social isolation and loneliness	Chronic kidney disease incidence, mortality, and life expectancy	Social isolation and loneliness were associated with chronic kidney disease incidence, chronic kidney disease mortality, and reduced life expectancy.	Social isolation and loneliness were independently associated with elevated CKD incidence, CKD mortality, and reduced life expectancy.	Population-based cohort with incidence, mortality, and life-expectancy outcomes.
Matthews et al. 2024 [45]	Multi-cohort observational study	Early and mid-adulthood cohorts	Social isolation and loneliness	Inflammatory markers	Social isolation showed more consistent associations with inflammation than loneliness.	No single comparable clinical OR/HR was reported; socially isolated participants had higher suPAR levels, with more consistent associations for suPAR than CRP or IL-6.	Mechanistic multi-cohort biomarker study.
Vespa et al. 2023 [46]	Observational multimorbidity study	Older adults with multimorbidity	Loneliness	Quality of life	Loneliness was associated with poorer quality of life.	Loneliness and advancing age were associated with poorer quality of life.	Observational multimorbidity study; no OR/HR because the outcome was quality-of-life score.
Gao et al. 2025 [47]	Longitudinal cohort, England	Adults followed between 2004 and 2018	Chronic loneliness and isolation phenotypes	Functional impairment and mortality	Chronic social deficits were associated with functional impairment and mortality.	Chronic loneliness and isolation phenotypes predicted incident functional impairment and all-cause or cause-specific mortality.	Longitudinal cohort analysis.

Key Findings

Cardiovascular outcomes were the most frequently represented domain. Longitudinal evidence showed that loneliness, social isolation, or broader social disconnection was associated with several cardiovascular outcomes, including incident cardiovascular disease, heart disease, stroke, cardiovascular mortality, cardiac dysfunction, and broader morbidity and mortality outcomes [21,22,27,28,37,38,41]. In individual studies, Xie et al. [27] reported increased incident cardiovascular disease among lonely middle-aged and older Chinese adults. Chen et al. [22] found that persistent loneliness and persistent social isolation were associated with a higher risk than transient exposure. Qin et al. [28] showed that social asymmetry, reflecting mismatch between subjective loneliness and objective isolation, was associated with morbidity and mortality.

For metabolic and cardiometabolic outcomes, the main findings involved diabetes, metabolic syndrome, cardiometabolic multimorbidity, non-alcoholic fatty liver disease, and malnutrition risk. Loneliness was associated with incident type 2 diabetes, metabolic syndrome, cardiometabolic multimorbidity, incident non-alcoholic fatty liver disease, and malnutrition risk among older diabetic outpatients [17,30,31,40-42]. Among individuals with metabolic syndrome, loneliness and social isolation were associated with all-cause and cause-specific mortality, particularly when both exposures were present [25].

Respiratory findings mainly involved chronic obstructive pulmonary disease mortality and adult-onset asthma. Social isolation was associated with higher mortality among adults with chronic obstructive pulmonary disease, partly mediated by systemic inflammatory markers [23]. Loneliness was associated with adult-onset asthma in a UK Biobank cohort, whereas social isolation was not significant after full adjustment [26]. For kidney disease, loneliness was associated with incident chronic kidney disease among adults with diabetes, while both loneliness and social isolation were associated with chronic kidney disease incidence, chronic kidney disease-related mortality, and reduced life expectancy in a population-based cohort [43,44]. Qualitative evidence also showed that chronic kidney disease may restrict social participation and deepen loneliness and isolation [32].

Frailty, functional impairment, and quality-of-life findings supported the relevance of social disconnection to geriatric and multimorbidity outcomes. Loneliness was associated with frailty among older adults in Singapore and Europe [36,39]. Chronic loneliness and isolation phenotypes were associated with functional impairment and mortality [47]. Among older adults with multimorbidity, loneliness was associated with poorer quality of life [46].

Behavioral and biological pathway studies provided plausible explanatory evidence. Loneliness and social isolation were associated with physical inactivity, smoking-related behaviors, poorer diet, weaker preventive behavior, and lower medication adherence [20,33,34]. Biological studies linked social isolation or loneliness with blood pressure, C-reactive protein, fibrinogen, systemic inflammatory ratios, and other inflammatory markers [20,23,29,45]. Overall, the evidence suggests that social disconnection is a clinically relevant social determinant of chronic disease-related outcomes among adults in community, primary care, outpatient, and population-based settings.

Risk of Bias

Risk of bias varied by study design, exposure measurement, outcome ascertainment, and confounding control. Large prospective cohorts and registry-linked studies were generally judged as low or low-to-moderate risk because they used longitudinal designs, larger samples, and stronger outcome ascertainment. Cross-sectional studies, small clinical samples, qualitative studies, and mechanistic studies were generally judged to be at moderate or high risk due to limited temporality, small sample sizes, indirect exposure measurement, or residual confounding. Cohort and registry-linked studies assessed using the Newcastle-Ottawa Scale are summarized in Table 4.

**Table 4:** Newcastle-Ottawa Scale assessment of cohort and registry-linked observational studies. The Newcastle-Ottawa Scale was used for cohort and registry-linked observational studies. Risk judgments were categorized as low, low to moderate, moderate, moderate to high, or high according to selection, comparability, exposure or outcome ascertainment, follow-up, confounding control, and suitability for causal interpretation.

Study	Overall risk of bias	Main concerns	Interpretation
Valtorta et al. 2018 [21]	Low to moderate	Self-reported loneliness or isolation, possible residual confounding	Longitudinal design supports a cardiovascular temporal association.
Chen et al. 2024 [22]	Low	Attrition and self-reported exposure were possible	Strong prospective evidence because repeated exposure patterns and clinical outcomes were assessed.
Yue et al. 2026 [23]	Low to moderate	Chronic obstructive pulmonary disease severity confounding and social isolation measurement limitations	Strong mortality evidence with inflammatory mediation analysis.
Cao et al. 2026 [24]	Low to moderate	Broad social disconnection exposure and residual confounding	Large cohort supports association with structural cardiovascular disease.
Zhang et al. 2026 [25]	Low	Residual confounding and self-reported social exposure were possible	Strong mortality evidence in a metabolic syndrome population.
Zhang et al. 2026 [26]	Low	Exposure self-report and Mendelian randomization assumptions require cautious interpretation	Stronger inference because cohort analysis was supported by Mendelian randomization.
Xie et al. 2026 [27]	Low to moderate	Single-item loneliness and possible self-reported cardiovascular outcomes	Good longitudinal evidence from Chinese adults.
Qin et al. 2026 [28]	Low	Constructed residual exposure was complex, and residual confounding was possible	Strong evidence due to long follow-up and linked morbidity and mortality outcomes.
Wang and Wang 2026 [30]	Low to moderate	Single-item loneliness and possible cohort measurement differences	Strong longitudinal evidence across two East Asian cohorts.
Hackett et al. 2020 [31]	Low	Self-reported loneliness and attrition were possible	Strong diabetes evidence due to diabetes-free baseline and extensive adjustment.
Naito et al. 2021 [35]	Low to moderate	Exposure definition may vary across countries, residual confounding possible	Strong global evidence because of multinational prospective design.
Freak-Poli et al. 2021 [37]	Low to moderate	Differences between support, isolation, and loneliness complicate interpretation	Strong cardiovascular evidence, especially for structural social disconnection.
Christiansen et al. 2021 [38]	Low	Residual confounding possible despite registry linkage	Strong chronic disease evidence due to prospective registry-linked design.
Ye et al. 2024 [39]	Low to moderate	Possible conceptual overlap between social frailty and loneliness	Good longitudinal evidence for frailty.
Xiao et al. 2024 [41]	Low	Self-reported exposure and residual confounding possible	Strong cardiometabolic multimorbidity evidence from a large prospective cohort.
Miao et al. 2025 [42]	Low	Residual confounding and mediation assumptions were possible	Strong non-alcoholic fatty liver disease evidence with prospective design and mediation analysis.
Tang et al. 2024 [43]	Low to moderate	Confounding by diabetes severity possible	Useful renal evidence in high-risk diabetic adults.
Guo et al. 2026 [44]	Low	Residual confounding possible	Strong chronic kidney disease evidence due to population-based cohort and hard outcomes.
Gao et al. 2025 [47]	Low	Self-reported social exposure and attrition were possible	Strong evidence because chronic exposure phenotypes were linked with functional and mortality outcomes.

Cross-sectional, panel, mechanistic, and other non-randomized observational studies assessed using Joanna Briggs Institute appraisal tools are summarized in Table 5.

**Table 5:** Joanna Briggs Institute assessment of cross-sectional, panel, mechanistic, and other observational studies. JBI: Joanna Briggs Institute. Joanna Briggs Institute appraisal tools were used for cross-sectional, panel, mechanistic, and other non-randomized observational studies where the Newcastle-Ottawa Scale was not the primary tool. Risk judgments considered clarity of inclusion criteria, exposure measurement, outcome measurement, identification and handling of confounding, statistical analysis, temporality, and relevance to the review question.

Study	Overall risk of bias	Main concerns	Interpretation
Ida et al. 2023 [17]	Moderate	Small outpatient sample, cross-sectional design, possible residual confounding	Useful diabetes-related evidence, but temporality cannot be confirmed.
Kurpas et al. 2016 [18]	Moderate	Social relationships were an indirect exposure, and confounding was possible	Useful respiratory care evidence, but weaker for direct loneliness or isolation.
Hu et al. 2021 [19]	Moderate	Cross-sectional design, subgroup differences, possible residual confounding	Supports the distinction between social constructs and cardiovascular risk factors.
Shankar et al. 2011 [20]	Moderate	Some associations were cross-sectional, and exposures were self-reported	Strong behavioral and biomarker pathway evidence, but causal inference is limited.
Kelechi et al. 2026 [29]	Moderate to high	Specialized chronic wound population, likely small sample, complex biological modeling	Useful mechanistic evidence, but not strong for clinical effect estimation.
Yamaguchi et al. 2020 [33]	High	Very small sample, cross-sectional design, limited adjustment	Useful mainly as behavioral pathway evidence.
Lu et al. 2020 [34]	Moderate	Cross-sectional mediation model cannot confirm direction	Relevant adherence evidence, but causal pathway remains uncertain.
Ge et al. 2022 [36]	Moderate	Time-varying confounding, possible overlap between frailty and loneliness	Useful longitudinal frailty evidence.
Whisman 2010 [40]	Moderate	Cross-sectional design, limited causal inference	Useful metabolic syndrome evidence, but temporality is unclear.
Matthews et al. 2024 [45]	Moderate	Biomarker timing and cohort heterogeneity	Supports biological plausibility, but pathways are heterogeneous.
Vespa et al. 2023 [46]	Moderate	Observational design, quality-of-life subjectivity, residual confounding	Useful patient-centered multimorbidity evidence.

Discussion

Summary of Main Findings

This systematic review found that social disconnection, including loneliness and social isolation, was associated with a wide range of chronic disease-related outcomes among adults in community, primary care, outpatient, registry-based, and population-based settings. The most central findings involved cardiovascular disease, type 2 diabetes, cardiometabolic multimorbidity, chronic kidney disease, frailty, functional impairment, and mortality. Additional evidence was identified for respiratory outcomes, malnutrition risk among older adults with diabetes, medication adherence, preventive health behavior, quality of life in multimorbidity, and inflammatory markers. Overall, the findings suggest that social disconnection is a clinically relevant social determinant associated with chronic disease-related outcomes, although causal inference remains limited by the observational nature and heterogeneity of the included evidence.

Domain-Specific Outcomes

Cardiovascular outcomes were among the most consistently represented domains in this review. Several longitudinal and population-based studies reported associations of loneliness, social isolation, or broader social disconnection with incident cardiovascular disease, heart disease, stroke, and cardiac dysfunction [21,22,27,28,37,38,41]. Other studies linked these exposures with cardiovascular mortality and broader morbidity and mortality outcomes [21,22,27,28,37,38,41]. Chen et al. [22] reported that persistent loneliness and persistent social isolation were more strongly associated with adverse cardiovascular and mortality outcomes than transient exposure, suggesting that the duration and stability of social disconnection may be clinically relevant. Xie et al. [27] reported increased incident cardiovascular disease among lonely middle-aged and older Chinese adults, while Qin et al. [28] suggested that mismatch between subjective loneliness and objective isolation may identify individuals with increased morbidity and mortality risk. These findings support the importance of assessing both perceived and structural dimensions of social connection in cardiovascular risk research.

Metabolic and cardiometabolic findings were mainly reported for type 2 diabetes, metabolic syndrome, cardiometabolic multimorbidity, non-alcoholic fatty liver disease, and malnutrition risk. Loneliness was associated with incident type 2 diabetes, metabolic syndrome, cardiometabolic multimorbidity, non-alcoholic fatty liver disease, and malnutrition risk among older diabetic outpatients [17,30,31,40-42]. Hackett et al. [31] reported that loneliness was associated with incident type 2 diabetes after adjustment for demographic, behavioral, metabolic, depressive, and social factors. Wang and Wang [30] found that persistent loneliness was associated with cardiometabolic multimorbidity across two East Asian cohorts, while Xiao et al. [41] reported associations of social isolation and loneliness with progression from single cardiometabolic disease to multimorbidity. These findings suggest that loneliness and social isolation may be related to metabolic risk through overlapping behavioral, psychological, inflammatory, and self-management pathways.

Respiratory and renal findings indicate that social disconnection may be associated with both disease incidence and prognosis. Yue et al. [23] reported that social isolation was associated with higher mortality among adults with chronic obstructive pulmonary disease, with systemic inflammatory markers partly mediating this association. Zhang et al. [26] found that loneliness was associated with adult-onset asthma, whereas social isolation was not significant after full adjustment. For kidney disease, Tang et al. [43] reported that loneliness was associated with incident chronic kidney disease among adults with diabetes. Guo et al. [44] reported that loneliness and social isolation were associated with chronic kidney disease incidence, chronic kidney disease-related mortality, and reduced life expectancy. Sluiter et al. [32] added qualitative context by describing how chronic kidney disease may restrict social participation and intensify loneliness and isolation. Together, these findings highlight a possible bidirectional relationship between social disconnection and chronic illness.

Frailty, functional impairment, and quality-of-life findings further support the relevance of social disconnection in aging and multimorbidity. Loneliness was associated with frailty among older adults in Singapore and Europe [36,39]. Gao et al. [47] reported that chronic loneliness and isolation phenotypes were associated with functional impairment and mortality, suggesting that long-term social deficits may be related to declining independence over time. Vespa et al. [46] also reported an association between loneliness and poorer quality of life among older adults with multimorbidity. These findings are clinically relevant because frailty, functional limitation, and quality of life are central outcomes in chronic disease care, especially among older adults.

Potential Mechanisms and Pathways

The included studies suggest several plausible pathways linking social disconnection with chronic disease-related outcomes. Behavioral pathways included physical inactivity, smoking-related behaviors, poorer diet, weaker preventive behavior, and lower medication adherence [20,33,34]. Biological pathways included blood pressure, C-reactive protein, fibrinogen, systemic inflammatory ratios, and other inflammatory markers [20,23,29,45]. These findings support a biopsychosocial interpretation in which loneliness and social isolation may be associated with chronic disease outcomes through stress biology, inflammation, reduced self-care, and loss of practical or emotional support. However, these pathways are unlikely to be uniform across all disease domains. Inflammatory pathways may be more relevant in chronic obstructive pulmonary disease, chronic wounds, and cardiometabolic disease, while self-management and adherence pathways may be more relevant in diabetes, chronic kidney disease, and multimorbidity.

Loneliness and Social Isolation as Distinct Constructs

A key interpretation from this review is that loneliness and social isolation are related but not interchangeable. Loneliness is a subjective emotional experience, whereas social isolation reflects an objective or structural lack of contact, participation, or network connection. Several studies showed that these constructs had different associations with outcomes [19,20,26,37,43,45]. This distinction matters because interventions for loneliness may require emotional, psychological, or relational approaches, whereas interventions for social isolation may require structural strategies such as improving access, social participation, transportation, community engagement, and practical support.

Bidirectionality

The findings also highlight the possibility of bidirectionality. Social disconnection may be associated with chronic disease outcomes through behavioral, psychological, and biological pathways, but chronic disease may also increase loneliness and social isolation by limiting mobility, reducing independence, increasing treatment burden, and restricting participation in family or community life. This bidirectional relationship is especially relevant for chronic kidney disease, chronic respiratory disease, frailty, and multimorbidity, where symptoms, appointments, functional decline, and dependency may gradually narrow social networks. Therefore, associations observed in cross-sectional studies should be interpreted cautiously. Longitudinal studies provide stronger temporal evidence, but they still cannot fully exclude residual confounding or reverse causation.

Clinical and Public Health Implications

From a clinical and public health perspective, assessment of social disconnection may add value to chronic disease management. Screening for loneliness and social isolation in primary care, outpatient clinics, and community programs may help identify adults at higher risk of poor self-management, frailty, functional decline, disease progression, and mortality. This may be particularly relevant for older adults and patients with diabetes, chronic kidney disease, chronic respiratory disease, cardiometabolic disease, and multimorbidity. However, screening alone is unlikely to be sufficient unless linked to practical interventions, referral pathways, community resources, and follow-up systems.

Strengths

This review has several strengths. It included 31 studies across multiple countries, populations, settings, and chronic disease domains, allowing broad synthesis of the available evidence. Many included studies were large longitudinal or registry-linked cohorts, which strengthened interpretation of temporality between social disconnection and later chronic disease outcomes compared with cross-sectional evidence alone. The review also separated loneliness and social isolation instead of combining them into one broad exposure, allowing clearer interpretation of their potentially different roles. In addition, the inclusion of behavioral, biological, qualitative, and disease-specific evidence helped provide a broader understanding of how social disconnection may relate to chronic disease outcomes. However, this broad scope also contributed to substantial heterogeneity.

Limitations

Several limitations should be considered. The included studies varied in exposure measurement, outcome definition, follow-up duration, adjustment strategy, and effect reporting. Some studies used single-item loneliness measures, which may not fully capture the depth, duration, or context of loneliness. Cross-sectional studies could not establish temporality, and reverse causation remains possible because chronic disease can itself increase social disconnection. Residual confounding by depression, socioeconomic status, baseline disease severity, disability, functional limitation, healthcare access, and lifestyle factors may also remain. Because of heterogeneity in populations, exposures, outcomes, and effect measures, meta-analysis was not performed, and the findings were synthesized narratively. In addition, not all included studies reported comparable adjusted effect estimates, confidence intervals, P values, or covariate sets, which limited direct comparison of the magnitude and precision of associations across studies. Additional limitations include restriction to English-language articles, exclusion of grey literature and preprints, lack of prospective protocol registration, and absence of a formal GRADE certainty assessment. Although a PRISMA flow diagram was prepared, full-text exclusion reasons were summarized by category rather than reported individually for each excluded article, which may limit detailed reproducibility of the study-selection process.

Future Research

Future research should use standardized and repeated measures of loneliness and social isolation, distinguish subjective and objective social disconnection, and report disease-specific outcomes using comparable definitions. More longitudinal studies are needed in primary care and outpatient populations, especially among adults with established chronic disease. Future intervention studies should test whether reducing loneliness, increasing social participation, or strengthening practical support can improve chronic disease outcomes, self-management, functional status, quality of life, and survival.

## Conclusions

Social disconnection, including loneliness and social isolation, appears to be associated with multiple chronic disease-related outcomes among adults in community, primary care, outpatient, registry-based, and population-based settings. The reviewed evidence suggests relevance across cardiovascular, metabolic, renal, respiratory, functional, quality-of-life, morbidity, and mortality outcomes. However, because the evidence was heterogeneous and largely observational, causal interpretation should remain cautious.

Loneliness and social isolation should be assessed as related but distinct constructs in chronic disease research. Future longitudinal and intervention studies are needed to determine whether improving social connection can support chronic disease self-management, functional outcomes, disease progression, and survival.

## References

[1] World Health Organization (2026). From Loneliness to Social Connection: Charting a Path to Healthier Societies. Report of the WHO Commission on Social Connection. https://www.who.int/publications/i/item/978240112360.

[2] World Health Organization (2026). Noncommunicable Diseases. https://www.who.int/news-room/fact-sheets/detail/noncommunicable-diseases.

[3] National Academies of Sciences, Engineering Engineering, and Medicine (2020). Social Isolation and Loneliness in Older Adults: Opportunities for the Health Care System.

[4] Holt-Lunstad J, Smith TB, Layton JB (2010). Social relationships and mortality risk: a meta-analytic review. PLoS Med.

[5] Holt-Lunstad J, Smith TB, Baker M, Harris T, Stephenson D (2015). Loneliness and social isolation as risk factors for mortality: a meta-analytic review. Perspect Psychol Sci.

[6] Valtorta NK, Kanaan M, Gilbody S, Ronzi S, Hanratty B (2016). Loneliness and social isolation as risk factors for coronary heart disease and stroke: systematic review and meta-analysis of longitudinal observational studies. Heart.

[7] Leigh-Hunt N, Bagguley D, Bash K, Turner V, Turnbull S, Valtorta N, Caan W (2017). An overview of systematic reviews on the public health consequences of social isolation and loneliness. Public Health.

[8] Hawkley LC, Cacioppo JT (2010). Loneliness matters: a theoretical and empirical review of consequences and mechanisms. Ann Behav Med.

[9] Cacioppo JT, Hawkley LC (2009). Perceived social isolation and cognition. Trends Cogn Sci.

[10] Courtin E, Knapp M (2017). Social isolation, loneliness and health in old age: a scoping review. Health Soc Care Community.

[11] Luo Y, Hawkley LC, Waite LJ, Cacioppo JT (2012). Loneliness, health, and mortality in old age: a national longitudinal study. Soc Sci Med.

[12] Steptoe A, Shankar A, Demakakos P, Wardle J (2013). Social isolation, loneliness, and all-cause mortality in older men and women. Proc Natl Acad Sci U S A.

[13] Page MJ, McKenzie JE, Bossuyt PM (2021). The PRISMA 2020 statement: an updated guideline for reporting systematic reviews. BMJ.

[14] Wells GA, Shea B, O’Connell D (2026). The Newcastle-Ottawa Scale for Assessing the Quality of Nonrandomised Studies in Meta-Analyses. https://ohri.ca/en/who-we-are/core-facilities-and-platforms/ottawa-methods-centre/newcastle-ottawa-scale.

[15] Joanna Briggs Institute (2026). JBI Critical Appraisal Tools. https://jbi.global/critical-appraisal-tools..

[16] Critical Appraisal Skills Programme (2026). CASP Qualitative Studies Checklist. https://casp-uk.net/casp-tools-checklists/qualitative-studies-checklist.

[17] Ida S, Kaneko R, Imataka K, Okubo K, Azuma K, Murata K (2023). Loneliness is associated with the risk of low nutrition in elderly diabetic patients. Nihon Ronen Igakkai Zasshi.

[18] Kurpas D, Szwamel K, Mroczek B (2016). Importance of social relationships in patients with chronic respiratory diseases. Adv Exp Med Biol.

[19] Hu J, Fitzgerald SM, Owen AJ (2021). Social isolation, social support, loneliness and cardiovascular disease risk factors: a cross-sectional study among older adults. Int J Geriatr Psychiatry.

[20] Shankar A, McMunn A, Banks J, Steptoe A (2011). Loneliness, social isolation, and behavioral and biological health indicators in older adults. Health Psychol.

[21] Valtorta NK, Kanaan M, Gilbody S, Hanratty B (2018). Loneliness, social isolation and risk of cardiovascular disease in the English Longitudinal Study of Ageing. Eur J Prev Cardiol.

[22] Chen Y, Xue H, Nie Y (2024). Evaluation of changes in social isolation and loneliness with incident cardiovascular events and mortality. J Epidemiol Glob Health.

[23] Yue X, Yao Q, Wang S (2026). Social isolation and mortality in chronic obstructive pulmonary disease: evidence from 1999 to 2018 NHANES with mediation by systemic inflammation. Medicine (Baltimore).

[24] Cao C, Wei C, Gong W, Yu B, Fang Z, Zhou S, Zhu Z (2026). Social disconnection, genetic risk, and the incidence of degenerative valvular heart disease: a population-based cohort study. J Am Heart Assoc.

[25] Zhang Y, Wang L, Bai J (2026). Association of social isolation and loneliness with all-cause and cause-specific mortality in individuals with metabolic syndrome: a prospective study. J Affect Disord.

[26] Zhang J, Li Y, Sun X (2026). Associations of social isolation and loneliness with the risk of adult-onset asthma: a prospective cohort and mendelian randomization study. Chron Respir Dis.

[27] Xie B, Fang Y, Lin F, Yang M, Liang Y, Liu B (2026). Association of loneliness with incident cardiovascular diseases in middle-aged and older Chinese adults. PLoS One.

[28] Qin P, Graham EK, Ong AD, Steptoe A (2026). Social asymmetry and risk of morbidity and mortality. JAMA Netw Open.

[29] Kelechi TJ, Cole SW, Theeke L (2026). Use of a social genomics model to explore loneliness and systemic inflammation in an adult population with chronic lower extremity ulcers. Adv Skin Wound Care.

[30] Wang M, Wang H (2026). Change in loneliness and subsequent cardiometabolic multimorbidity among middle-aged and older adults: results from two East Asian prospective cohorts. Aging Clin Exp Res.

[31] Hackett RA, Hudson JL, Chilcot J (2020). Loneliness and type 2 diabetes incidence: findings from the English Longitudinal Study of Ageing. Diabetologia.

[32] Sluiter A, Cazzolli R, Jaure A (2024). Experiences of social isolation and loneliness in chronic kidney disease: a secondary qualitative analysis. Clin J Am Soc Nephrol.

[33] Yamaguchi Y, Yamada M, Hapsari ED, Matsuo H (2020). The influence of social isolation on the preventive behaviors for non-communicable diseases in community-dwelling older adults in Japan. Int J Environ Res Public Health.

[34] Lu J, Zhang N, Mao D, Wang Y, Wang X (2020). How social isolation and loneliness effect medication adherence among elderly with chronic diseases: an integrated theory and validated cross-sectional study. Arch Gerontol Geriatr.

[35] Naito R, Leong DP, Bangdiwala SI (2021). Impact of social isolation on mortality and morbidity in 20 high-income, middle-income and low-income countries in five continents. BMJ Glob Health.

[36] Ge L, Yap CW, Heng BH (2022). Associations of social isolation, social participation, and loneliness with frailty in older adults in Singapore: a panel data analysis. BMC Geriatr.

[37] Freak-Poli R, Ryan J, Neumann JT (2021). Social isolation, social support and loneliness as predictors of cardiovascular disease incidence and mortality. BMC Geriatr.

[38] Christiansen J, Lund R, Qualter P, Andersen CM, Pedersen SS, Lasgaard M (2021). Loneliness, social isolation, and chronic disease outcomes. Ann Behav Med.

[39] Ye L, Bally E, Korenhof SA (2024). The association between loneliness and frailty among community-dwelling older adults in five European countries: a longitudinal study. Age Ageing.

[40] Whisman MA (2010). Loneliness and the metabolic syndrome in a population-based sample of middle-aged and older adults. Health Psychol.

[41] Xiao Z, Li J, Luo Y, Yang L, Zhang G, Cheng X, Bai Y (2024). Social isolation and loneliness with risk of cardiometabolic multimorbidity: a prospective cohort study from UK Biobank. iScience.

[42] Miao Y, Kong X, Zhao B, Fang F, Chai J, Huang J (2025). Loneliness and social isolation with risk of incident non-alcoholic fatty liver disease, UK biobank 2006 to 2022. Health Data Sci.

[43] Tang R, Zhou J, Wang X, Ma H, Li X, Heianza Y, Qi L (2024). Loneliness, social isolation and incident chronic kidney disease among patients with diabetes. Gen Psychiatr.

[44] Guo Y, Li X, Liu Y (2026). Association of social isolation and loneliness with the incidence and life expectancy of chronic kidney disease: a population-based cohort study. Int J Surg.

[45] Matthews T, Rasmussen LJ, Ambler A (2024). Social isolation, loneliness, and inflammation: a multi-cohort investigation in early and mid-adulthood. Brain Behav Immun.

[46] Vespa A, Spatuzzi R, Fabbietti P (2023). Association between sense of loneliness and quality of life in older adults with multimorbidity. Int J Environ Res Public Health.

[47] Gao Q, Steptoe A, Fancourt D (2025). Chronic loneliness and isolation phenotypes, incident functional impairment and mortality in England between 2004 and 2018. Nat Ment Health.

